# A Topology-Based Stereo Matching Method for One Shot 3D Measurement Using Coded Spot-Array Structured Light

**DOI:** 10.3390/s21196444

**Published:** 2021-09-27

**Authors:** Junhui Mei, Xiao Yang, Zhenxin Wang, Xiaobo Chen, Juntong Xi

**Affiliations:** 1 School of Mechanical Engineering, Shanghai Jiao Tong University, Shanghai 200240, China; junhui_mei@126.com (J.M.); yangxiao1982@sjtu.edu.cn (X.Y.); honghuimail@sjtu.edu.cn (Z.W.); jtxi@sjtu.edu.cn (J.X.); 2 Shanghai Key Laboratory of Advanced Manufacturing Environment, Shanghai 200240, China; 3 Shanghai AllyNav Technology Corp., Ltd., Shanghai 201700, China; 4 State Key Laboratory of Mechanical System and Vibration, Shanghai 200240, China

**Keywords:** stereo matching, active stereo, single pattern projection, spot array

## Abstract

In this paper, a topology-based stereo matching method for 3D measurement using a single pattern of coded spot-array structured light is proposed. The pattern of spot array is designed with a central reference ring spot, and each spot in the pattern can be uniquely coded with the row and column indexes according to the predefined topological search path. A method using rectangle templates to find the encoded spots in the captured images is proposed in the case where coding spots are missing, and an interpolation method is also proposed for rebuilding the missing spots. Experimental results demonstrate that the proposed technique could exactly and uniquely decode each spot and establish the stereo matching relation successfully, which can be used to obtain three-dimensional (3D) reconstruction with a single-shot method.

## 1. Introduction

Non-contact optical measurement technologies are currently very popular and are extensively used in industrial inspection tasks due to their advantages of high efficiency, moderate accuracy and large measurement range compared with contact measurement technology. There exist many non-contact optical measurement technologies, such as triangulation, time of flight (TOF), interferometry and so on. Generally, the non-contact optical measurement technologies based on triangulation can be divided into two categories: stereo vision and structured light. Stereo vision realizes the 3D reconstruction of a point by “seeing” the point with two cameras at different perspectives. However, for the surfaces with no texture or little texture variation, the stereo matching process can be very difficult. Structured light technology can solve this problem by replacing one camera by a projector, which can generate artificial texture in order to ease the stereo matching process.

To obtain better stereo matching results, a lot of projection patterns of structured light are developed. Some review papers on this topic have been published [1,2,3,4,5]. In reference [4], Geng classified the structured light techniques into multiple-shot and single-shot categories. Furthermore, Geng divided single-shot techniques into three broad categories. Compared with the multiple-shot technique, the single-shot technique can perform a snapshot 3D measurement, which is very suitable for 3D reconstruction of moving targets. Up to now, the single-shot structured light technique is still a very hot research area [6,7,8,9,10]. Xu proposed a real-time 3D shape measurement approach based on one-shot structured light [6]. The method can satisfy the requirement of the online inspection of automotive production lines. To decode the correspondence of structured light, Zhang proposed a discontinuity-preserving matching method to improve the decoding of one-shot shape acquisition using regularized color [7]. Sagawa presented a grid-pattern based 3D reconstruction method, which can obtain a dense one-shot reconstruction by calculating dense phase information from a set of periodically encoded parallel lines [8]. However, the computational cost of this method is relatively high, which renders it unsuitable for real-time applications. Li designed a single striped pattern to measure the dense and accurate depth maps of 3D moving objects [9]. In this method, the De Bruijn sequence is used to encode the ID of each stripe in the pattern, and the epipolar segment is employed to eliminate periodic ambiguity. Garcia-Isais proposed a method to use a single composite fringe pattern containing three different frequencies to solve the problem of periodic ambiguity caused by surface discontinuities [10]. In this manner, the shape of objects having discontinuities or being spatially isolated can be reconstructed by projecting a single pattern. Since the Fourier method is employed, however, some information at the object edges or low modulation zones might be lost.

Although single-shot structured light techniques are very powerful in measurement efficiency, they still have a problem in obtaining accurate sensor calibration results because a simple and accurate calibration of a general projector is still a challenge compared with a camera calibration [11]. To overcome the disadvantage of structured light and simultaneously facilitate the stereo correspondence of stereovision, some researchers combined the two methods together [11,12,13,14,15,16,17,18,19,20]. Sun proposed a system that combined the technology of binocular stereo vision and multi-line structured light [12]. In this system, the time-multiplexing coding method was used and a sequence of patterns is projected. Pinto developed a system using stereo vision and fringe projection to measure large surfaces [13]. The system projects a sequence of sinusoidal fringes, in combination with a binary Graycode, onto the measurement surface. The absolute phase values are used for determining the correct 3D points of the measurement surface. Han proposed a method that combined the stereovision and phase shifting techniques [14]. This method can eliminate errors caused by inaccurate phase measurement. Three fringe patterns with a phase shift of π/3 are projected horizontally and then vertically. However, projecting too many patterns might consume a longer period of time, during which any movement of the object or the sensor would cause errors in 3D measurement. This prevents the method from measuring some workpiece, such as a hull plate, in the workshop where environmental factors such as vibration and movement often play a negative role.

Reducing the number of projection pattern is a very efficient method for improving measurement speed. Han used a visibility-modulated fringe pattern to further eliminate the need of the second fringe pattern [15]. With this new pattern, only three fringe patterns are required. To reduce the number of gray code images in the projection sequence, Burchardt proposed a method that used the restriction of the valid measuring volume and epipolar constraints to reduce the valid area for proper point correspondences [16]. Lohry also adopted three fringe patterns, which were modified to encode the quality map for efficient and accurate stereo matching [11]. In order to avoid global phase unwrapping, a random pattern is used to match corresponding phase points between two images roughly. After determining the coarse disparity map, a refined disparity map is further obtained by the local wrapped phase. Generally, in order to obtain the highest speed, the desired number of projection patterns might be one. Wang used a single pattern of coded stripes to measure the diameter of a hot large forging [17]. A spatial coded strategy is employed to characterize each stripe with a unique index. Thus, the stereo correspondence is established according to the stripe index and the corresponding epipolar line. Additionally, random illumination of structured light has been introduced into stereovision [18,19,20]. The random illumination-based methods can obtain a single-shot dense 3D reconstruction. In reference [19], the feature-based approach is introduced into stereo correspondence in order to preserve a low error rate. In reference [20], a method of disparity updating based on temporal consistency was further proposed in order to improve the speed of 3D measurement. In the above-mentioned methods [17,18,19,20], the epipolar constraint is employed in order to find correspondence points between two cameras. However, the stereo correspondence might suffer from errors caused by epipolar lines.

Sometimes, a dense 3D reconstruction is not necessary for some special objects, such as a large hull plate. Generally, the discrete sampling points can be used for representing a free-form surface. There are some methods that measure a free-form surface by reconstructing sample points on the surface [13,21,22]. In reference [13], the density of sample points is freely definable. However, this method requires projecting multiple patterns for a single measurement. In reference [21,22], however, only a few sample points, such as five or nine spots, are reconstructed for a single measurement.

In our previous work [23], an onsite inspection sensor based on active binocular stereo vison is proposed. This sensor has two cameras for composing a binocular stereo vision system and a projector to project structured light. The measurement principles, system development and system calibration are described in detail. A pattern with circular spot array is used to generate feature points onto the surface to be measured. Additionally, each spot is encoded with a gray code by projecting a sequence of gray code patterns onto the surface. With the extra projection of gray code patterns, effects of occlusion, discontinuous and depth step on spot stereo matching can be well eliminated. However, it is not very efficient since several extra gray code patterns are projected during one measurement, thus rendering this method unsuitable for onsite measurement of formed hull plates, especially in a workshop environment with radon vibration. In this paper, based on our previous work, a method that combines only one single-shot pattern of spot-array structured light and stereo vision is used to measure continuous free-form surfaces. A pattern of circular spot array with a marking referenced ring spot is designed. The circular spots in the pattern are row and column aligned. With the help of a digital projector, the spot density can also be adjusted according to the shape of a measured surface. Additionally, an algorithm for coding each spot with a unique 2D index is also developed. With the unique 2D index, the circular spot in the left and right camera can be matched directly without using the epipolar constraint. Then, the 3D reconstruction of the spot is calculated by triangulation. As only a single pattern is used, the proposed system is expected to be immune to vibration and disturbance problems encountered in an onsite inspection environment. It is very suitable for the onsite measurement of hull plates, because there are many vibrations in the formation workshop of hull plates. Moreover, the proposed method is very cost effective and easy to implement.

The rests of the paper are organized as follows. Section 2 illustrates the coding principle. Experiments are performed in Section 3. Finally, Section 4 summarizes the paper.

## 2. Coding Principle

**Figure 1:**
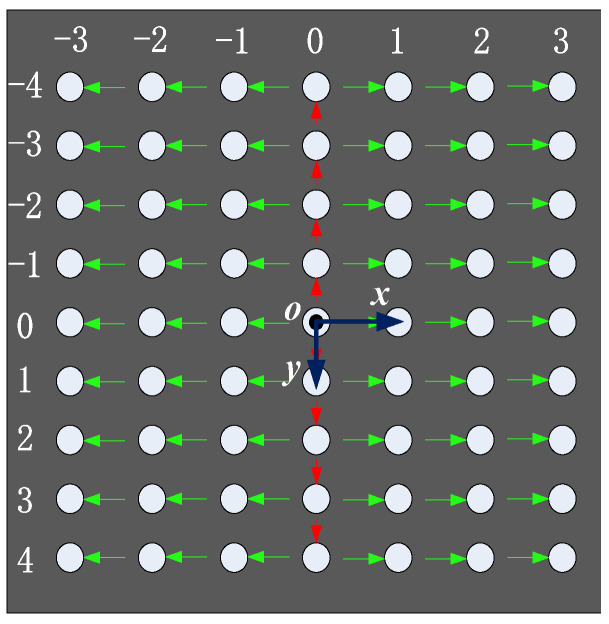
Coding process of a spot array.

In order to illustrate the coding principle, a circular spot array of 9 rows × 7 columns is generated as an example shown in Figure 1. In the array, a special ring is used as reference center. According to the reference ring, a coordinate system can be built as shown in Figure 1, where the origin is the center of the reference ring, and the x and y axes are parallel to the image row and column, respectively. Then, the row and column indexes are used to code each spot in this coordinate system. In order to code each spot accurately, a coding process based on a topological relationship is defined in this paper. The coding process is divided into two steps, as shown in Figure 1. The first step is to code the spot located at origin and then to code the others along the positive and negative directions of y axis, respectively, which are denoted by red arrows. The second step is to code the spots in each row, and the coding sequences are denoted by green arrows.

### 2.1. Extracting Center Points of Spots

Before coding a spot array, the first step that should be performed is to detect the spots and extract their centers. The shape of the spot is generally a circle. For a circle, we can obtain the following:

(1) $$s=\frac{l^{2}}{4\pi }$$

where s is the area, and l is the perimeter. According to Equation (1), roundness can be deduced as follows.

(2) $$c=\frac{l^{2}}{4\pi s}$$

**Figure 2:**
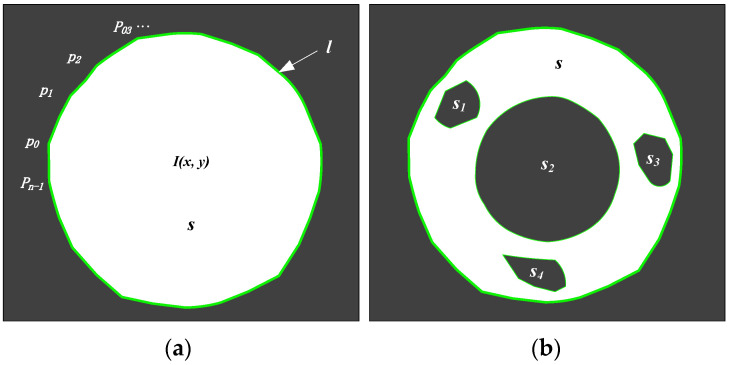
Connected components in an image: (**a**) a circle; (**b**) a ring.

For an ideal circle, c should be 1. Therefore, two roundness thresholds of $C_{min}$ and $C_{max}$ can be used to exclude some outliers:

(3) $$C_{min}\leq c\leq C_{max}$$

where $C_{min}$ and $C_{max}$ are the minimum and maximum roundness, respectively. In order to calculate the roundness of a spot, the area and the perimeter should be known according to Equation (2). For a circular spot in an image shown in Figure 2a, the area and the external contour perimeter can be obtained by using the following equations:

(4) $$s=\underset{x}{\sum }\underset{y}{\sum }I\left(x,y\right)$$

(5) $$l=\|p_{n-1}p_{0}{\|}_{2}+\overset{n-2}{\underset{i=0}{\sum }}\|p_{i}p_{i+1}{\|}_{2}$$

where *x* and *y* are the pixel coordinates; I(x,y) is the value after image binarization, which equals 1 inside the spot area and equals 0 outside the spot area; n is the number of pixels on the external contour; $p_{i}$ depicts the *i*th pixel on the external contour; and $\|p_{i}p_{i+1}{\|}_{2}$ represents the distance between the two adjacent pixels $p_{i}$ and $p_{i+1}$ on the spot area contour. Additionally, if the area threshold is considered, the outliers can be further excluded by using the following inequality:

(6) $$S_{min}\leq s\leq S_{max}$$

where $S_{min}$ and $S_{max}$ are the minimum and maximum area. In summary, if a spot does not satisfy the inequalities (3) and (6), it can be considered as an outlier.

From the above analysis, it is known that the reference ring plays a very important role in coding a spot array. However, it might not satisfy the inequalities (3) and (6). In order to avoid excluding the reference ring from the spot array, some improvements are made for the spot detection algorithm. Without loss of generality, a ring with some noise blobs are considered, which is shown in Figure 2b. Obviously, the area surrounded by the external contour can be figured out by:

(7) $$S=s+\underset{i=1}{\sum }s_{i}$$

where s is the area of the ring’s effective connected component; $s_{i}$ is the area surrounded by the *i*th internal contour. Furthermore, the roundness of the ring’s external contour can be expressed as:

(8) $$C=\frac{l_{0}{}^{2}}{4\pi S}$$

where $l_{0}$ is the perimeter of the ring’s external contour. For the reference ring, C and S should satisfy the inequalities (3) and (6), because the ring’s external contour is the same as other circles. Then, the ring should be distinguished from the other circles. It is well known that a ring in general is composed by two concentric circles, which are a large one and a small one. The area ratio of the two circles for the reference ring is designed to be 0.6 when the spot-array pattern is designed. Therefore, we can detect the small circle to find the reference ring. For the reference ring, it should have an internal circle satisfying the following expression:

(9) $$\{\begin{array}{c} 0.6S_{min}\leq s_{i}\leq 0.6S_{max}\\ C_{min}\leq c_{i}\leq C_{max} \end{array}/$$

where $s_{i}$ is the area surrounded by the *i*th internal contour and $c_{i}$ is the roundness of the *i*th internal contour. Therefore, if a spot has some internal contours and only one of the internal contours satisfies the following inequality (9), it can be taken as the reference ring.

After the detection of the spot array, the centers of the spots should be extracted. The method proposed in our previous work [24] is employed to extract the centers of the spots in this paper.

### 2.2. Coding Spots with Row and Column Indexes

From Figure 1, it is known that the reference ring firstly should be coded with row and column indexes of (0, 0). Then, each spot can be coded one by one along a fixed search path. Therefore, for a coded spot in a search path, it is very crucial to accurately find the next uncoded spot in the same search path. In this paper, a method that uses rectangular templates to search the spots along a search path is proposed. Generally, in the design pattern, the spots are arranged in straight lines parallel to the image row or column. For this case, rectangular templates might be extended along the straight line, which renders the search process simple. However, the spots would be arranged in curves when the pattern is projected onto a free-form surface. For this case, the extension direction of rectangular templates should be adjusted to the tangents of curves. In order to illustrate the proposed method in detail, coding processes of spots arranged in a straight line and a curve are described in the following, respectively.

#### 2.2.1. Spots Arranged in a Straight Line

**Figure 3:**
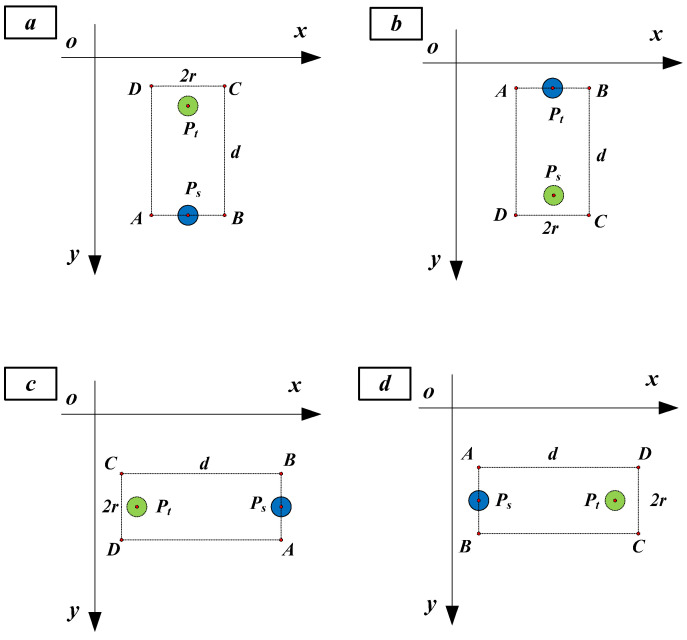
Four search directions: (**a**) up, (**b**) down, (**c**) left and (**d**) right.

In a design pattern, for a given coded spot $p_{s}$ ($x_{0}$, $y_{0}$), there are four search directions of an uncoded spot $p_{t}$ (x, y), as shown in Figure 3. The four search directions are up, down, left and right, which are illustrated in Figure 3a–d, respectively.

Then, the four rectangle templates are generated as follows. First, the longer side of the rectangle denoted by d should be extended along the search direction. Second, the shorter side of the rectangle denoted by 2r should be vertical with respect to the search direction, and one of the short sides takes $p_{s}$ as the center. Third, as the search direction is parallel to the image row or column, the coordinates of the four corners of the rectangle denoted by *A*, *B*, *C* and *D* can be easily determined, as shown in Table 1. In Table 1, it can be judged whether $p_{t}$ is within the rectangle *ABCD*. If $p_{t}$ is outside *ABCD*, d and r will be enlarged until *ABCD* contains it. Suppose point $p_{s}$ is coded with row and column indexes of ($i_{0}$, $j_{0}$) and point $p_{t}$ is within the *ABCD*, the row and column indexes of $p_{t}$ can then be coded, as shown in Table 1, according to the search direction v and the number of intervals n.

**Figure 4:**
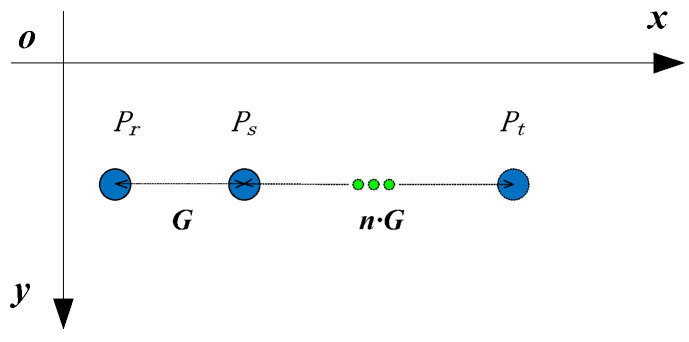
Missing spots arranged in a straight line.

For a design pattern, n might be one. However, for noise effects such as ambient illumination and non-uniform reflectivity of the measurement surface, some spots in the captured image of the spot array projected on the surface cannot be detected in practical application. Therefore, in order to obtain accurate row and column indexes, the number of intervals needs to be calculated if there are some missing spots in a search path. To succinctly illustrate this issue, three spots are arranged in a straight line, which are $p_{r}$, $p_{s}$ and $p_{t}$ from left to right as shown in Figure 4. In Figure 4, $p_{r}$ and $p_{s}$ are actually two adjacent spots while there are n−1 missing spots between $p_{s}$ and $p_{t}$. If the distance between $p_{r}$ and $p_{s}$ is G, which is called unit distance in this paper, then the distance between $p_{s}$ and $p_{t}$ in theory should be n⋅G. In practice, as the unit distance might change at the different areas of a spot array projected on a measured surface, G is calculated as follows. First, find the four neighborhood spots of the reference ring, which are up, down, left and right, by the method shown in Figure 3. Second, calculate the distance between each of the four neighborhood spots and the reference ring. Then, compare the four distances and choose the middle distance as the initial value of G. Third, renew the value of G by the distance between two local adjacent spots in a search path.

Suppose that the row and column indexes of $P_{r}$, $P_{s}$ and $P_{t}$ are ($I_{r}$, $J_{r}$), ($I_{s}$, $J_{s}$) and ($I_{t}$, $J_{t}$), respectively. For $P_{r}$ and $P_{s}$, the following can be deduced.

(10) $$\{\begin{array}{c} I_{s}=I_{r}\\ J_{s}=J_{r}+1 \end{array}$$

For $P_{s}$ and $P_{t}$, the following can be deduced.

(11) $$\{\begin{array}{c} I_{t}=I_{s}\\ J_{t}=J_{s}+n\\ n=\frac{\left|P_{s}P_{t}\right|}{G} \end{array}$$

Then, it can be observed that Equation (10) is in fact a special case of Equation (11), when $n=\frac{\left|P_{r}P_{s}\right|}{G}=\frac{G}{G=1}$. Therefore, for spots arranged in a straight line, the number of intervals between the coded and uncoded points and, moreover, the row and column indexes of the uncoded point can be determined by Equation (11), if the unit distance and the distance between the coded and uncoded points are obtained. Finally, for spots arranged in a straight line, the row and column indexes are coded, as shown in Table 1.

#### 2.2.2. Spots Arranged in a Curve

**Figure 5:**
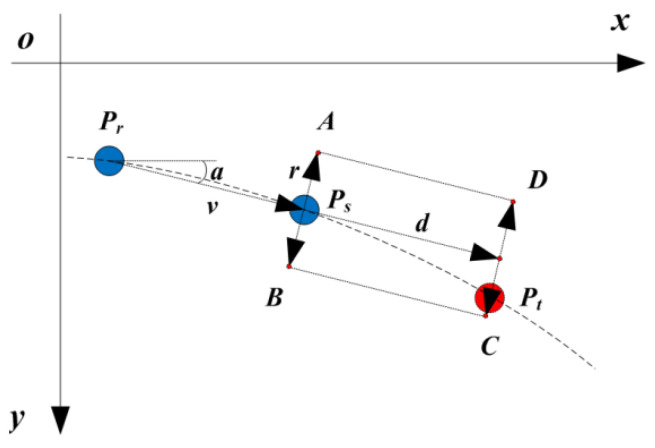
The search process of an uncoded spot in a curve.

For spots arranged in a curve, it is unsuitable to use the method proposed for coding spots arranged in a straight line. This is because the search path of a curve might have a different tangent at each spot, which is different from the search path of a straight line. Therefore, in order to accurately and quickly find the uncoded spot, the search direction should be along the tangent at each spot of the search path. Suppose there are three spots, $P_{r}$, $P_{s}$ and $P_{t}$, arranged in a curve from left to right. The three points are shown in Figure 5, where $P_{r}$ and $P_{s}$ are two coded points with known indexes of row and column, while $P_{t}$ is an uncoded point.

To find $P_{t}$, the following steps are performed. Firstly, $P_{s}$ is chosen as the search starting point. Secondly, the vector $\vec{P_{r}P_{s}}$ is calculated. Then, a normalized vector v can be determined: $v=\frac{{\vec{P_{r}P}}_{s}}{\left|\vec{P_{r}P_{s}}\right|}=\left(cosa,sina\right)$, where a is the angle between the v and x axis. Thirdly, take the vector v as the search direction, and a search rectangle template *ABCD* with length of d and width of 2r can be generated as shown in Figure 5. If the pixel coordinates of $P_{s}$ is ($x_{0}$, $y_{0}$), then the pixel coordinates of the four corners of the rectangle template can be deduced, respectively, as in the following.

(12) $$\{\begin{array}{c} x_{A}=x_{0}+rsina\\ y_{A}=y_{0}-rcosa \end{array}$$

(13) $$\{\begin{array}{c} x_{B}=x_{0}-rsina\\ y_{B}=y_{0}+rcosa \end{array}$$

(14) $$\{\begin{array}{c} x_{C}=x_{0}+dcosa-rsina\\ y_{C}=y_{0}+dsina+rcosa \end{array}$$

(15) $$\{\begin{array}{c} x_{D}=x_{0}+dcosa+rsina\\ y_{D}=y_{0}+dsina-rcosa \end{array}$$

Generally, the search direction used for generating the rectangle *ABCD* is not parallel to the x or y axis for a curved search path. Therefore, the criteria listed in Table 1 are not applicable for judging whether $P_{t}$ (*x*, *y*) is within *ABCD* for this case. In order to overcome this shortcoming, a more general criterion that $P_{t}$ is within *ABCD* is given as follows.

(16) $$\{\begin{array}{c} \left(\vec{AP_{t}}\times \vec{AD}\right)\left(\vec{BP_{t}}\times \vec{BC}\right)\leq 0\\ \left(\vec{BP_{t}}\times \vec{BA}\right)\left(\vec{CP_{t}}\times \vec{CD}\right)\leq 0 \end{array}$$

When $P_{t}$ is found by the rectangle template, the row and column indexes of $P_{t}$ can be determined by Table 1 according to the search direction.

#### 2.2.3. Image Coordinates Reconstruction of Missing Spots

For missing spots, their image coordinates should be reconstructed before they are coded with row and column indexes. As the true image coordinates of missing spots in general are unable to be detected in a captured image, estimation methods of image coordinates for missing spots arranged in a straight line and a curve are proposed, respectively, in this paper. For missing spots arranged in a straight line, we use the case shown in Figure 4 to illustrate the image coordinates estimation method for missing spots. It can be deduced that there are n−1 missing spots between $p_{s}$ and $p_{t}$. For missing spots arranged in a straight line, their image coordinates can be calculated by linear interpolation. Supposing that image coordinates $p_{s}$ and $p_{t}$ are $\left(x_{s},y_{s}\right)$ and $\left(x_{t},y_{t}\right)$, and row and column indexes $p_{s}$ and $p_{t}$ are $\left(I_{s},J_{s}\right)$ and $\left(I_{t},J_{t}\right)$, we can reconstruct the image coordinates of the *i*th missing spot $p_{i}\left(x_{i},y_{i}\right)$ as follows.

(17) $$\{\begin{array}{c} x_{i}=x_{s}+\frac{i}{n}\left(x_{t}-x_{s}\right)\\ y_{i}=y_{s}+\frac{i}{n}\left(y_{t}-y_{s}\right) \end{array}$$

The row and column indexes of $p_{i}\left(I_{i},J_{i}\right)$ can be determined by the following.

(18) $$\{\begin{array}{c} I_{i}=I_{s}=I_{t}\\ J_{i}=J_{s}+i \end{array}$$

From the above, it is known that the search direction is correct in Figure 4. For the other three search directions, we can still use Equation (17) to calculate the image coordinates of missing spots. However, Equation (18) could not be used to calculate the row and column indexes of missing spots for the other three directions and is only suitable for the search direction of right. In fact, for the *i*th missing spot, we can determine its row and column indexes according to Table 1 if the search direction is given.

**Figure 6:**
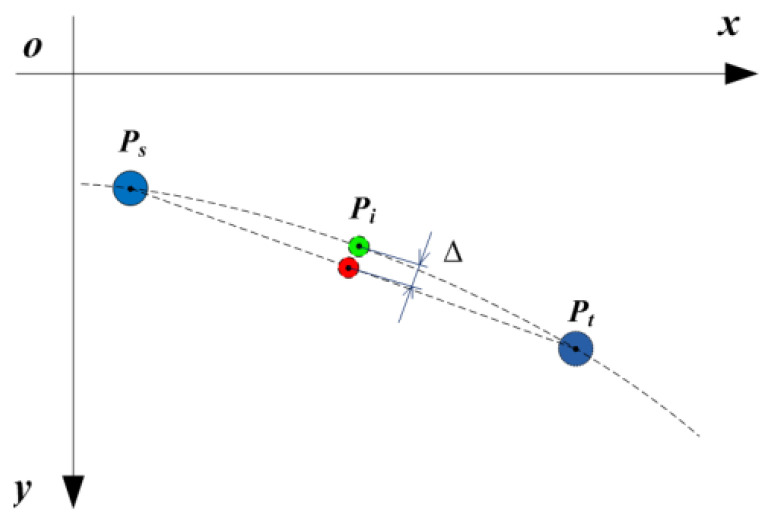
The calculation error of missing spots in a curve by linear interpolation.

Compared with in a straight line, the case that missing spots are in a curve is more general. As spots are not arranged in a straight line, the linear interpolation is not suitable for calculating the image coordinates of these missing spots, and it could result in a calculation error, as shown in Figure 6. In Figure 6, $p_{s}$ and $p_{t}$ are two coded spots for which their row and column indexes are known, while $p_{i}$ is a missing spot between $p_{s}$ and $p_{t}$. The green spot is the theoretical image coordinates of $p_{i}$, and the red spots are the ones calculated by the linear interpolation method. It is clearly observed that there is a deviation between these two spots.

**Figure 7:**
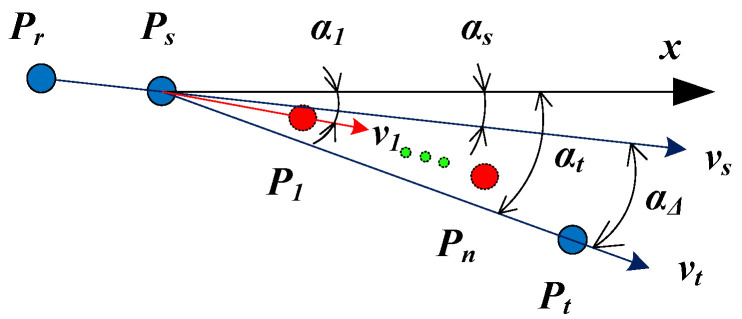
The interpolation of missing spots in a curve.

In order to reduce the calculation error of the linear interpolation method, a new interpolation method of missing spots in a curve is presented. The method is more accurate than the linear interpolation method. Moreover, it is efficient and simple to be executed. Suppose that there are n missing spots between $P_{s}$ and $P_{t}$, which are denoted by $P_{1}$, $P_{2}$, …, $P_{n}$, respectively, as shown in Figure 7.

In Figure 7, $v_{s}=\vec{P_{r}P_{s}}=\left(x_{vs},y_{vs}\right)$; $v_{t}=\vec{P_{s}P_{t}}=\left(x_{vt},y_{vt}\right)$; $a_{s}$ is the angle between $v_{s}$ and x axes; $a_{t}$ is the angle between $v_{t}$ and x axes; and $a_{\Delta }$ is the angle between $v_{s}$ and $v_{t}$. First of all, the missing spot $P_{1}$ is interpolated as an example to explain the interpolation method. If the pixel coordinates of $P_{1}$ are ($x_{1}$, $y_{1}$), then $v_{1}=\vec{P_{s}P_{1}}$ can be obtained. Supposing $a_{1}$ denotes the angle between $v_{1}$ and x axes, we can approximately calculate it by using the following equations.

(19) $$a_{1}=a_{s}+a_{\Delta }/n$$

(20) $$a_{\Delta }=a_{t}-a_{s}$$

Moreover, if the ambiguity of arccosine in the range of (0, 2π) is considered, $a_{s}$ and $a_{t}$ can be calculated as follows.

(21) $$a_{s}=\{\begin{array}{cc} arcos(\frac{x_{vs}}{\left|v_{s}\right|}) & y_{vs}\geq 0\\ -arcos(\frac{x_{vs}}{\left|v_{s}\right|}) & y_{vs}<0 \end{array}$$

(22) $$a_{t}=\{\begin{array}{cc} arcos(\frac{x_{vt}}{\left|v_{t}\right|}) & y_{vt}\geq 0\\ -arcos(\frac{x_{vt}}{\left|v_{t}\right|}) & y_{vt}<0 \end{array}$$

According to Equations (21) and (22), it can be deduced that $-\pi <a_{s}<\pi$ and $-\pi <a_{t}<\pi$. Then, according to Equation (20), $-2\pi <a_{\Delta }<2\pi$ can be obtained. However, considering the physical meaning of the angle between two vectors, $a_{\Delta }$ should be in a range of (−π, π). By employing the cycle of 2π, Equation (20) can be corrected as follows.

(23) $$a_{\Delta }=\{\begin{array}{cc} a_{t}-a_{s} & -\pi <a_{t}-a_{s}<\pi \\ a_{t}-a_{s}-2\pi & \pi <a_{t}-a_{s}<2\pi \\ a_{t}-a_{s}+2\pi & -2\pi <a_{t}-a_{s}<-\pi \end{array}$$

Therefore, $a_{1}$ can be calculated according to Equations (19) and (21)–(23), and then the pixel coordinates of $P_{1}$ can be calculated according to the following.

(24) $$\{\begin{array}{c} x_{1}=x_{0}+\frac{\left|\vec{P_{s}P_{t}}\right|cos\left(a_{1}\right)}{n}\\ y_{1}=y_{0}+\frac{\left|\vec{P_{s}P_{t}}\right|sin\left(a_{1}\right)}{n} \end{array}$$

Then, $P_{1}$ can be used as a new starting point, and the pixel coordinates of $P_{2}$ can be obtained by repeating the above process. The above process can be continuously repeated until the last missing spot $P_{n}$ is determined.

## 3. Experiments

In order to verify the performance of the proposed coding method, some patterns of spot array generated by a computer are tested. Then, the proposed coding method is used for stereo matching, and two objects with continuous surfaces are measured by a developed active stereovision system. The details of the experiments are presented as follow.

### 3.1. Coding Simulations

**Figure 8:**
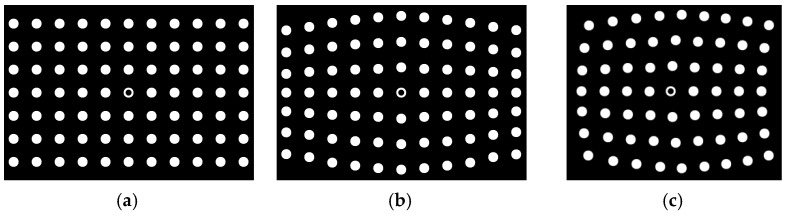
Complete spot-array patterns, (**a**) non-bend arrangement, (**b**) row-bend arrangement and (**c**) bidirection-bend arrangement.

**Figure 9:**
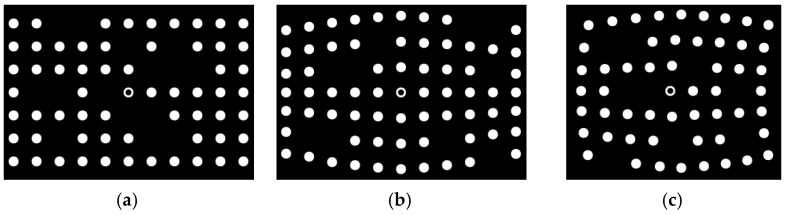
Incomplete spot-array patterns, (**a**) non-bend arrangement, (**b**) row-bend arrangement and (**c**) bidirection-bend arrangement.

**Figure 10:**
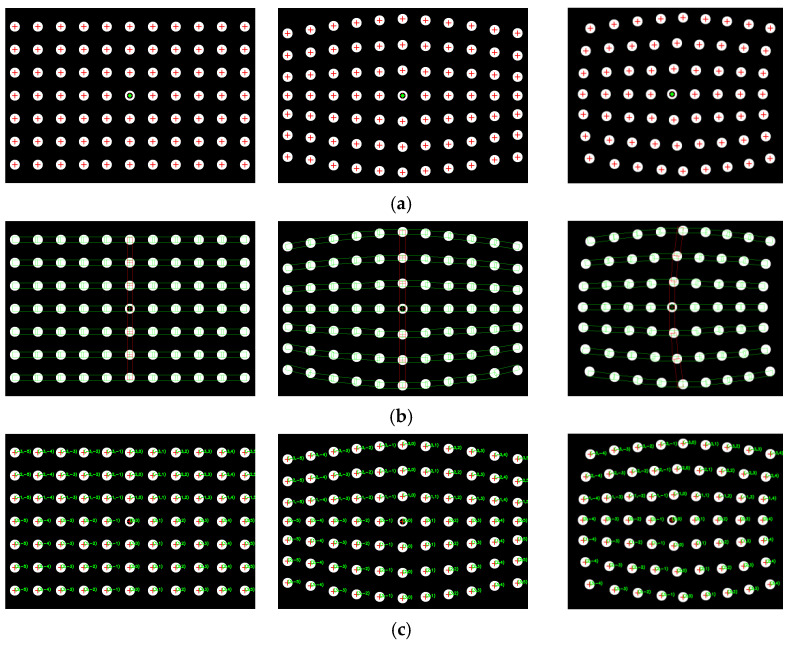
The coding results of complete spot-array patterns: (**a**) centers detection, (**b**) the display of rectangle templates and (**c**) the display of coding indexes.

**Figure 11:**
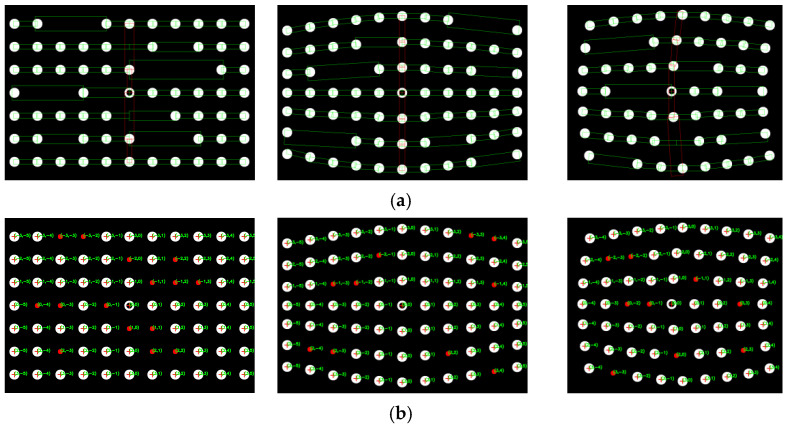
The coding results of incomplete spot-array patterns, (**a**) the display of rectangle templates and (**b**) the display of coding indexes.

Firstly, two sets of spot-array patterns are generated, as shown in Figure 8 and Figure 9. Compared with Figure 8, the spot-array patterns in Figure 9 are not complete, and there are some missing spots in these patterns. All of these patterns are coded by the algorithm, and the results are shown in Figure 10 and Figure 11, respectively.

Figure 10a shows that the proposed method can extract the centers of all spots. The reference ring for which its center is marked by a small green spot is successfully distinguished from circular spots. Figure 10b demonstrates that rectangle templates can accurately find spots along both the straight and curved search path. Finally, the spots in each pattern are exactly coded with row and column indexes, as shown in Figure 10c. Furthermore, for the case that there are some missing spots in the search path, the proposed algorithm can not only reconstruct the missing spots but also code them correctly, which are denoted by the red spots and demonstrated in Figure 11a,b.

### 3.2. 3D Measurements

**Figure 12:**
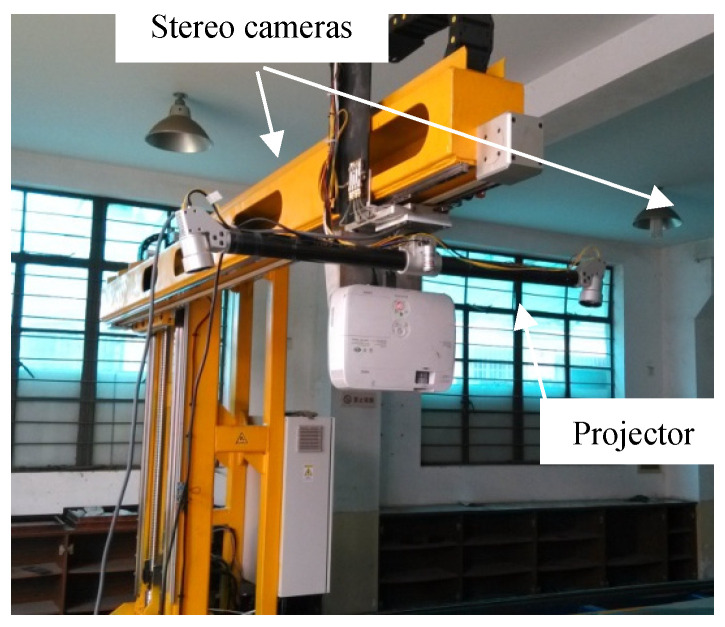
The experiment system of active binocular stereovision.

In order to verify the proposed topology-based stereo matching method for real 3D measurement, an experimental environment has been constructed, as shown in Figure 12. The constructed system includes two identical cameras and one projector. The camera resolution is 2048 × 1080 pixels, and the lens focal length is 8 mm. The projector resolution is 1024 × 768 pixels. The baseline length is about 1000 mm, and the working distance is about 1500 mm. The intrinsic and structure parameters of these two cameras are calibrated by the method proposed in Reference [25]. The projector does not need to be calibrated, because it is not involved in the triangulation process. The projector projects spot-array patterns onto the target surface, and the cameras capture pairs of images. Based on the proposed matching method, the corresponding spot centers can be established, which can be further 3D triangulated to reconstruct the surface shape.

**Figure 13:**
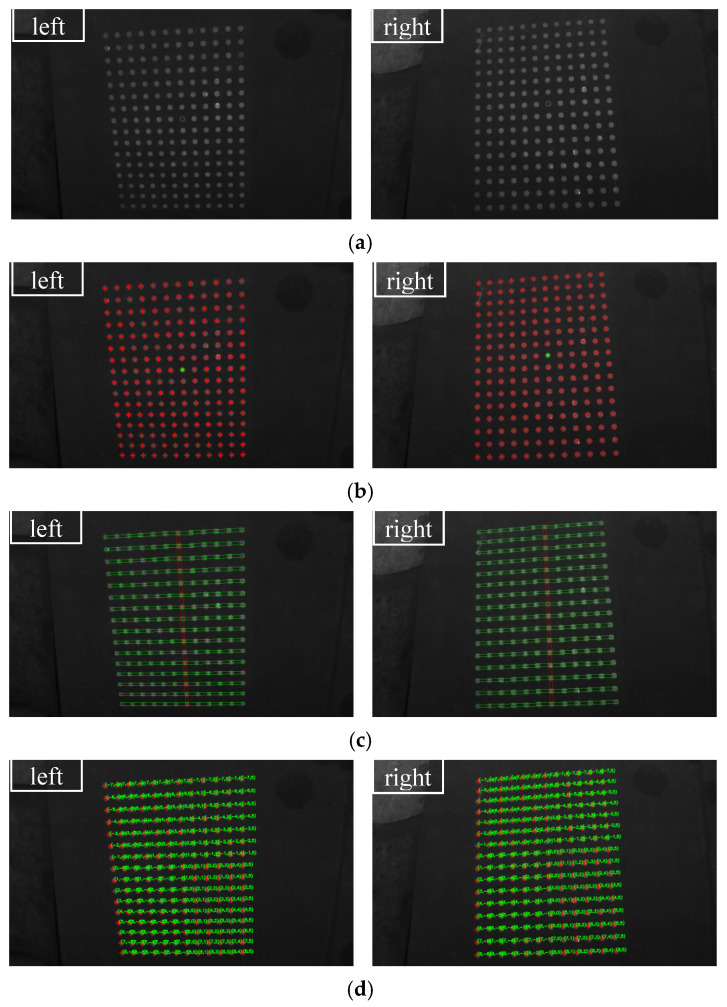
The coding process of the flat steel plate captured in the left and right cameras: (**a**) projection of a spot array on the surface, (**b**) centers detection, (**c**) rectangle templates display and (**d**) coding indexes display.

**Figure 14:**
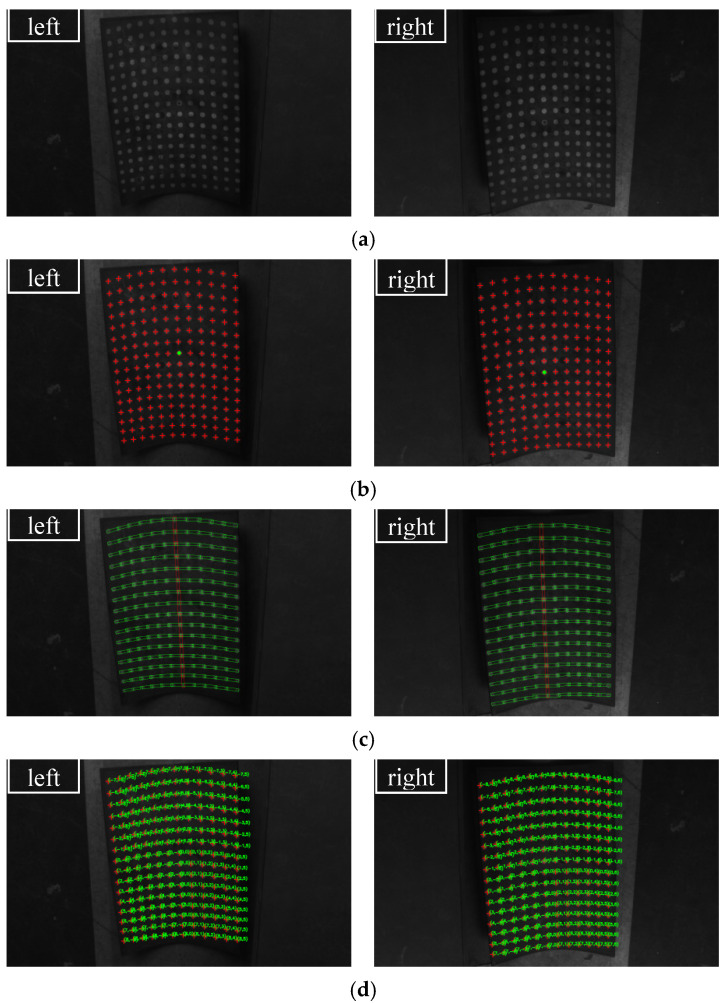
The coding process of the saddle-shaped plate captured in the left and right cameras: (**a**) projection of a spot array on the surface, (**b**) centers detection, (**c**) rectangle templates display and (**d**) coding indexes display.

**Figure 15:**
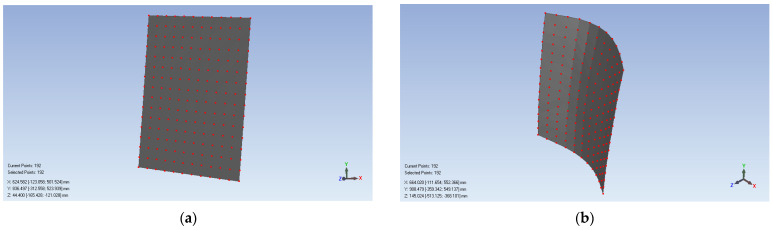
The 3D reconstruction results: (**a**) the flat steel plate and (**b**) the saddle-shaped hull plate.

We use this system to measure a flat steel plate and a saddle-shaped hull plate to verify the proposed topology-based stereo matching method. The flat steel plate has a size of 650 mm × 700 mm, and the saddle-shaped hull plate is a real part from a ship building factory. The measurement processes and results are shown in Figure 13 and Figure 14, respectively. Figure 13a and Figure 14b show the original captured scenes where the spot-array pattern is projected onto the plates. Figure 13b and Figure 14b show that the proposed method can successfully extract the reference ring spot from circular spots and extract the circular spot centers. Figure 13c and Figure 14c demonstrate that the rectangle templates can accurately find spots along both the straight and curved search paths. Then, these spots are exactly coded with row and column indexes, as shown in Figure 13d and Figure 14d. Finally, with the obtained coded indexes, the corresponding spots in the left and right camera images can be matched, and they can be further used to three-dimensionally reconstruct these spot centers. The 3D reconstruction results of the flat steel plate and the saddle-shaped hull plater are provided in Figure 15.

## 4. Conclusions

In order to avoid projector calibration and to improve measurement efficiency, a topology-based method of active stereo matching using a single pattern of spot array is proposed in this paper. The pattern of spot array is designed with a reference ring spot. Each spot in the pattern can be exactly and uniquely coded with the row and column indexes according to the topological search path. Coding the spots arranged in both a straight line and a curve is studied, and the issue of some missing spots in a search path is also analyzed. In order to solve these problems effectively, a method using rectangle templates to find uncoded spots is proposed. Moreover, an interpolation method that can rebuild missing spots is also developed. Compared with our previous work [23], the proposed method does not need to project extra gray code patterns; thus, this renders the measurement much faster and suitable for measuring a formed hull plate even in a workshop environment that normally has vibrations. Finally, computer simulations and real data testing show that the proposed method has a good performance. In addition to the ship building industry, the proposed topology-based stereo matching method for one-shot 3D measurement may contribute to real time measurement applications for the automotive industry or other application fields.

## Figures and Tables

**Table 1:** Coding spots arranged in a straight line.

Search Direction	Coordinates of the Four Corners of the Rectangle Template				Criteria That $P_{t}$ (*x*, *y*) Is within *ABCD*	Row and Column Indexes	
	*A*	*B*	*C*	*D*		*i*	*j*
Up	$x_{A}=x_{0}-r$ $y_{A}=y_{0}$	$x_{B}=x_{0}+r$ $y_{B}=y_{0}$	$x_{C}=x_{0}+r$ $y_{C}=y_{0}-d$	$x_{D}=x_{0}-r$ $y_{D}=y_{0}-d$	$x_{0}-r\leq x\leq x_{0}+r$ $y_{0}-d\leq y\leq v_{0}$	$i_{0}-n$	$j_{0}$
Down	$x_{A}=x_{0}+r$ $y_{A}=y_{0}$	$x_{B}=x_{0}-r$ $y_{B}=y_{0}$	$x_{C}=x_{0}-r$ $y_{C}=y_{0}+d$	$x_{D}=x_{0}+r$ $y_{D}=y_{0}+d$	$x_{0}-r\leq x\leq x_{0}+r$ $y_{0}\leq y\leq y_{0}+d$	$i_{0}+n$	$j_{0}$
Left	$x_{A}=x_{0}$ $y_{A}=y_{0}-r$	$x_{B}=x_{0}$ $y_{B}=y_{0}+r$	$x_{C}=x_{0}+d$ $y_{C}=y_{0}+r$	$x_{D}=x_{0}+d$ $y_{D}=y_{0}-r$	$x_{0}\leq x\leq x_{0}+d$ $y_{0}-r\leq y\leq y_{0}+r$	$i_{0}$	$j_{0}-n$
Right	$x_{A}=x_{0}$ $y_{A}=y_{0}+r$	$x_{B}=x_{0}$ $y_{B}=y_{0}-r$	$x_{C}=x_{0}-d$ $y_{C}=y_{0}-r$	$x_{D}=x_{0}-d$ $y_{D}=y_{0}+r$	$x_{0}-d\leq x\leq u_{0}$ $y_{0}-r\leq y\leq y_{0}+r$	$i_{0}$	$j_{0}+n$
